# Design and evaluation of a laboratory-based wheelchair castor testing protocol using community data

**DOI:** 10.1371/journal.pone.0226621

**Published:** 2020-01-10

**Authors:** Anand Mhatre, Norman Reese, Jon Pearlman

**Affiliations:** 1 Department of Rehabilitation Science and Technology, University of Pittsburgh, Pittsburgh, Pennsylvania, United States of America; 2 International Society of Wheelchair Professionals, Pittsburgh, Pennsylvania, United States of America; 3 Engineering & Engineering Technology, LeTourneau University, Longview, Texas, United States of America; University of Illinois at Urbana-Champaign, UNITED STATES

## Abstract

Wheelchair castors fail frequently causing physical, social and economic consequences for wheelchair users. These failures occur in spite of established wheelchair test methods and regulations, suggesting that the existing tests may not be sufficient to screen poorly designed castors. An expert stakeholder group, convened by the International Society of Wheelchair Professionals (ISWP), noted castor failures as a high priority and recommended that a new castor testing system should be developed. In a previous study, the effect of shock exposure on castor durability was studied. The current paper extends the previous work and focuses on the development of a castor testing protocol based on shock, corrosion and abrasion exposure data collected in the community. The testing protocol was applied to 8 different castor models tested under four conditions: shock, corrosion + shock, abrasion + shock and abrasion + corrosion + shock. For each model, a total of n = 8 samples were evaluated across the four conditions. Results demonstrate that corrosion and abrasion reduced castor durability between 13% to 100% depending on the model. Importantly, the inclusion of corrosion and abrasion resulted in changes in the failure modes for 75% of the tested models and two-thirds of the altered failure modes are associated with increased risk of injury for wheelchair users. These results suggest that corrosion and abrasion present in the community reduce castor durability, thus supporting their inclusion in the castor testing protocol and potentially other wheelchair standards.

## Introduction

Wheelchairs are known to fail frequently in a short period of time resulting in negative consequences for users [1–5]. Community-based data collection and maintenance studies conducted in United States, Scotland, Kenya, India and Mexico have reported breakdowns and part failures with castors, brakes, seats and tyres from a few weeks to two years of wheelchair use [1–3,5–11]. Failures are associated with significant adverse consequences, such as users being injured or stranded. For example, castor stem bolt fractures can cause the wheelchair to tip and the user to fall out of the wheelchair, which is the most common wheelchair injury [12,13]. Deferred maintenance can lead to a breakdown which can go unaddressed due to lack of repair and rehabilitation services, replacements parts and unavailability of skilled labour. Without a functional wheelchair, the user may have to stay in her bed and/or within the confines of her home which is associated with secondary health complications [14–18]. Hence, product failures lead to reduced user satisfaction and lower quality of life.

**Fig 1:**
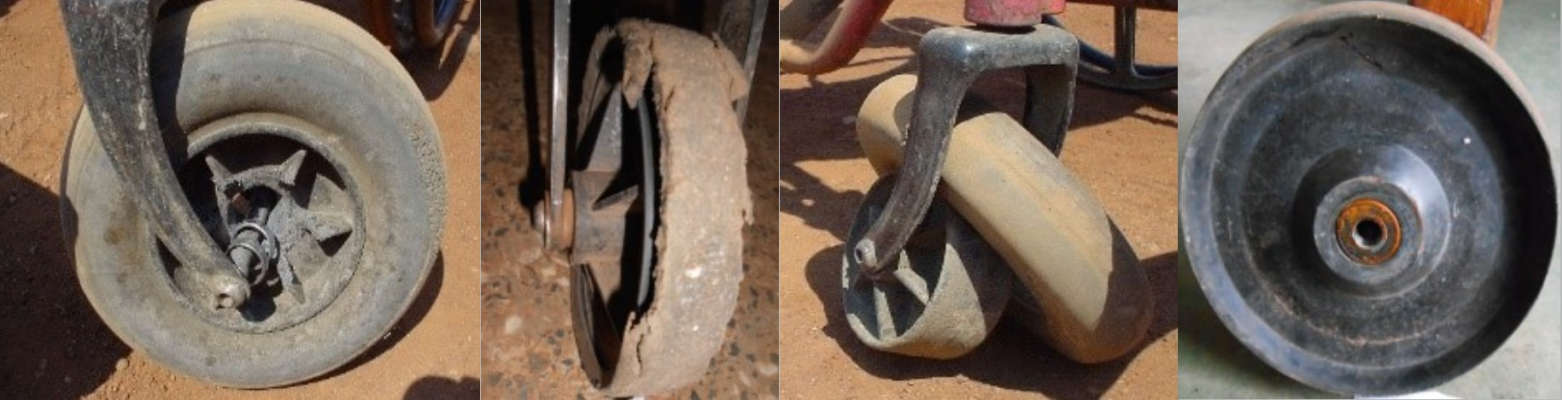
Castor axle bearing failure (left), tire cracking and worn-out (middle left), tire Roll-off (middle-right) and axle bearing corrosion (right).

Wheelchair castors are reported to experience frequent failures in the community due to different failure modes [1–3,5,9,19–21]. Studies documenting wheelchair incidents and repairs in the United States and Scotland found that nearly one-third of all wheelchair part failures are castor failures [9,20]. One study reported that castors and wheels contributed to 42% of the recorded wheelchair failures and 44% of adverse user consequences including physical, social and economic consequences [22]. Castors from wheelchair models designed for use in adverse environments with unpaved, rough terrains and hot and humid climates have been found to undergo multiple part failures in Kenya [1,5]. Locked and missing bearings, damaged bolts, fractured wheels and forks, worn-out tyres and missing fasteners are common castor failures (see Fig 1) [1,5,19,21]. In addition to community failures, castors are known to fail during laboratory-based ISO-7176 standards testing [23–35].

**Fig 2:**
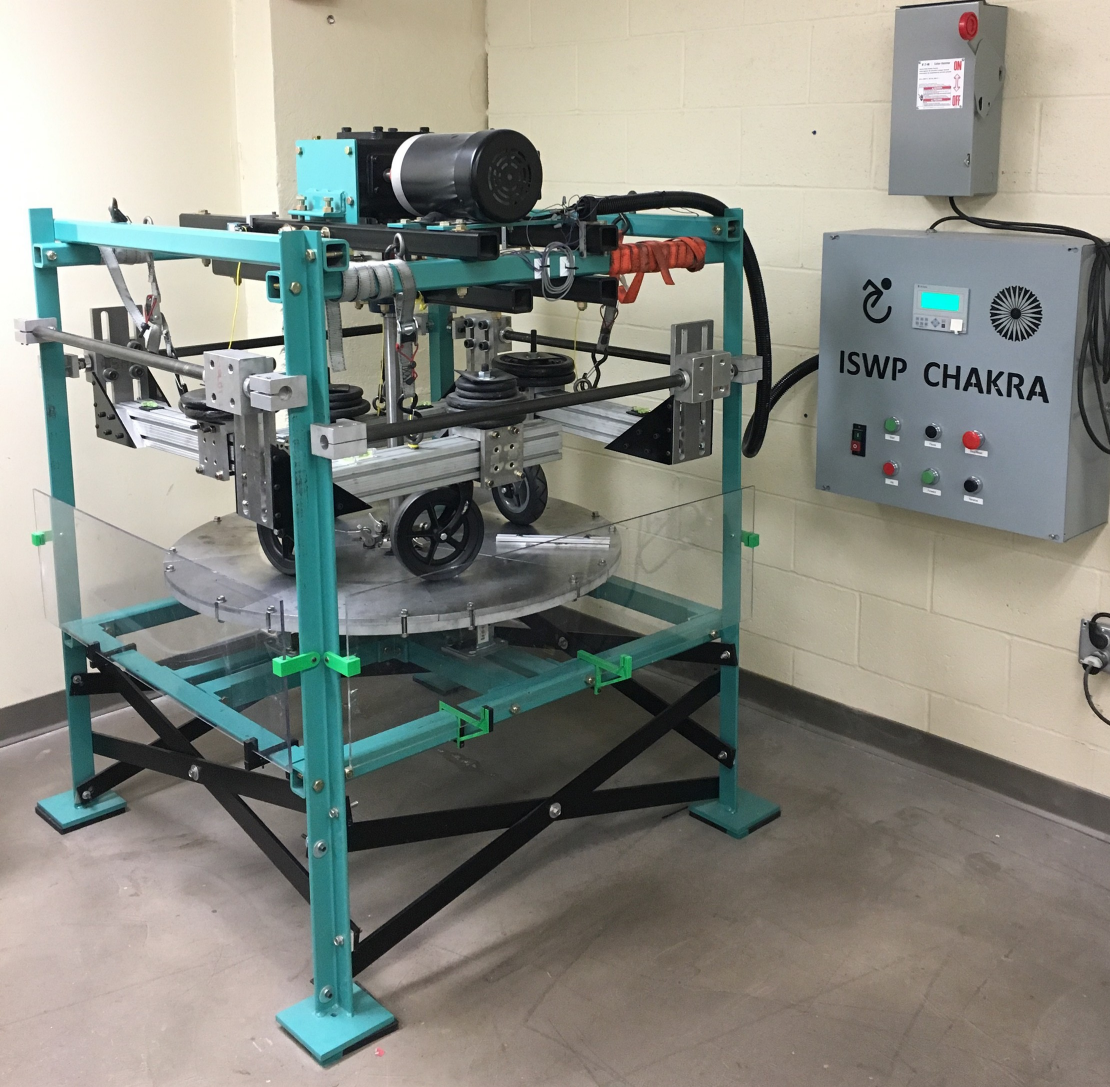
ISWP castor testing system.

To improve quality of castors and wheelchair products overall, several international organizations including the World Health Organization (WHO) and International Society of Wheelchair Professionals (ISWP) are promoting the provision of high-quality, appropriate wheelchairs [36–39]. Further, the WHO Guidelines on the provision of manual wheelchairs in less-resourced settings (LRS) recommend product testing specific to adverse environments witnessed in LRS and rural areas of resourced settings (RS) [40]. Based on this recommendation, the ISWP convened a Standards Working Group (ISWP-SWG) to guide wheelchair testing methods that could be proposed as amendments or extensions to the current suite of published ISO-7176 wheelchair standards that are more suitable for products used in urban areas in resourced settings [41,42]. The ISWP-SWG recommended that among several test methods that need to be developed or strengthened [41], castor testing is a high priority given their high rate of failure and associated injury risk. Based on these recommendations, the ISWP-SWG group has developed a laboratory-based castor testing system (see Fig 2) through an iterative design and expert review approach which was previously published [19], and is consistent with ISO/AWP 7176–32 [43]. Castor systems are suspended from arms on a turntable mimicking the way they are mounted on a wheelchair. The turntable accommodates triangular-shaped plates on which different types of obstacles and surfaces can be attached. Since shock is a primary environmental factor affecting castor durability, shock testing conditions similar to the ISO-7176 double drum fatigue testing machine were incorporated by attaching obstacles of 12.7mm thickness on diametrically opposite plates on the turntable [44,45]. Preliminary testing carried out with castors under shock testing condition exposed the weak links in the castor models. Castor stem bolts, bearings, forks and tyres experienced most failures. When compared to community failures reported by manufacturers, failures of stem bolt fracture, stem bearing fracture and tyre cracks matched anecdotally for some models, but approximately 60% of failure modes did not [19]. Manufacturers stated that wear-related failures caused by environmental factors such as corrosion and rough surfaces are common in the community and occur before fracture failures observed on the castor test. This feedback motivated and guided this work to develop a lab-based testing protocol that included shock, abrasion and corrosion using measurements of these conditions in the community.

## Research objective

This study describes the development and evaluation of a castor testing protocol which was carried out in two steps:

1. Development of a lab-based testing protocol that includes factors of shock, corrosion and abrasion based on exposures measured in the community.
2. Conducting lab-based castor testing using the testing protocol developed in [1] and measuring the relative impact of the individual testing factors (corrosion, shock and abrasion) on castor durability and failure mode.

## Materials and methods

**Fig 3:**
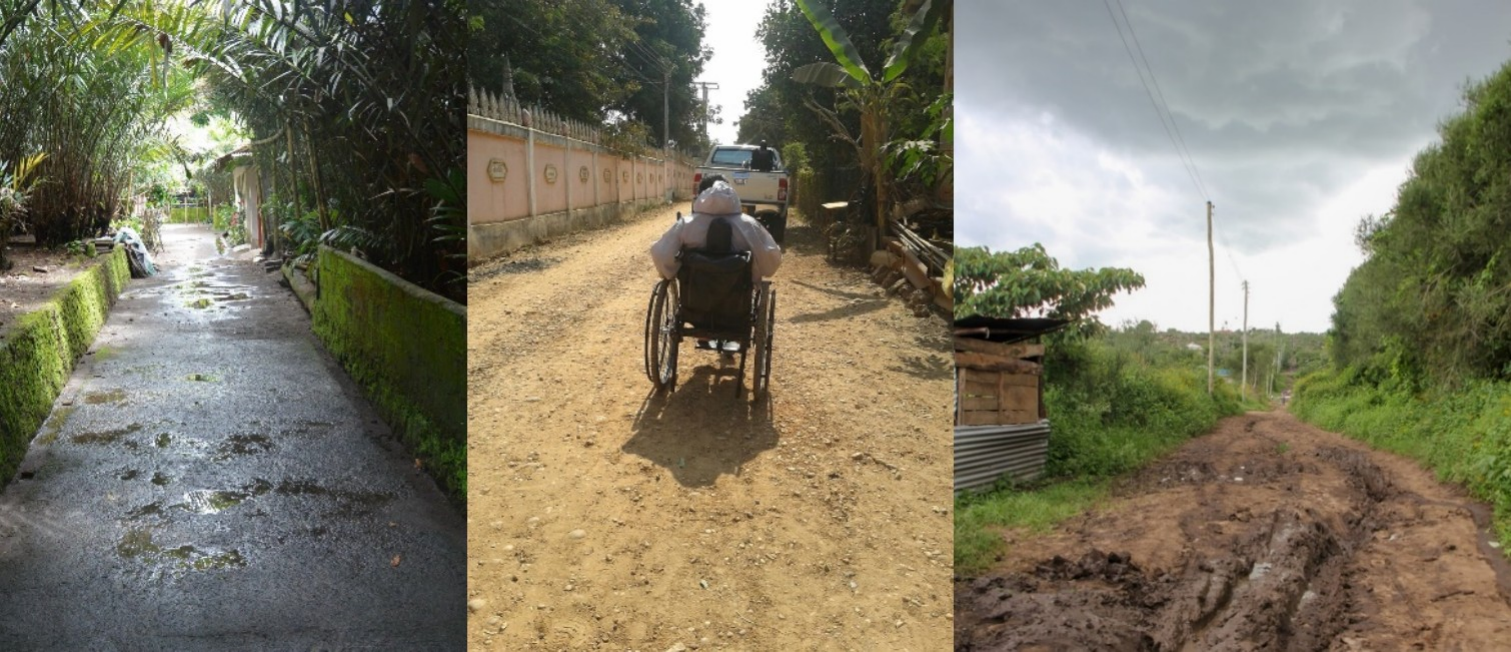
Examples of adverse conditions in LRS [46]. Picture on the right is reprinted under a CC BY license, with permission from Partners for Care, original copyright 2018.

To address the primary goal of developing the testing protocol, community data and failed castor samples collected by ISWP partners were used in this study. The co-author NR collected the data and materials as a part of another study that was approved by the Letourneau University’s Institutional Review Board. The shock data from the community was collected from Kenya as an environment typical of many LRS (Fig 3).

### Determining shock exposure based on measurements in the community

Shock exposure is caused by abrupt loads applied to the castors due to bumps, curbs, and drops. Failures due to shock are typically a yielded component after a major sudden load, or fatigue fracture after repetitive, fluctuating loads. In this study, shocks suffered by wheelchair castors were measured using accelerometers mounted on frames near castors.

**Fig 4:**
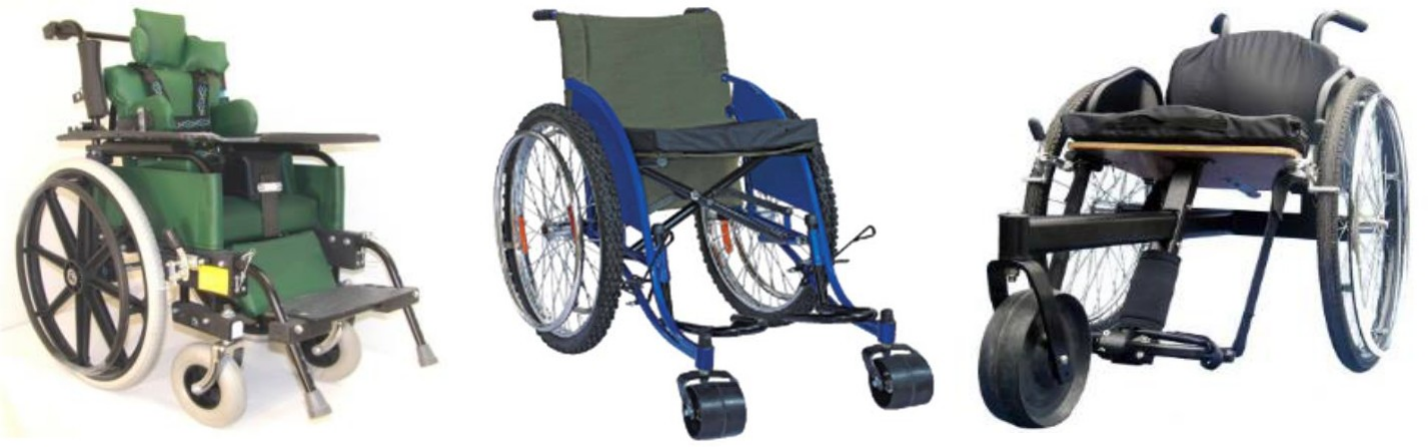
Hopehaven Kids Wheelchair (left), Whirlwind Roughrider (middle) and Motivation Rough Terrain Wheelchair (right) [41].

**Fig 5:**
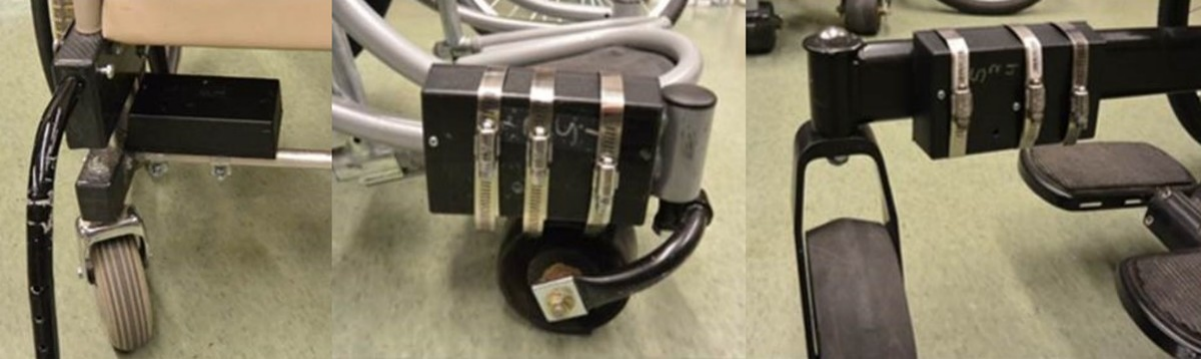
Accelerometers installed in a black box attached to the wheelchair frame.

Accelerations on castors of three wheelchair models commonly used in adverse environments–Whirlwind Roughrider (n = 1), Hopehaven Kids Wheelchair (n = 2) and Motivation Rough Terrain Wheelchair (n = 1) (Fig 4) were recorded in Kenya for eight days of use. The paediatric users were from a boarding school setting in a high-altitude area with uneven terrain and streets without pavement. Accelerometer model X16-1D with 3-axis, ±16g capability was used for recording acceleration data [47]. A sampling rate of 400Hz was selected as the fastest sampling rate available with low cost portable sensors capable of recording data over a period of days in the field without adding significant weight or tethers to the wheelchair. The sampling rate was suitable to prevent aliasing. The accelerometers were packed in a black box containing two D-size batteries to provide power for collecting data for a week and clamped above the castor hubs as shown in Fig 5. Once the study was completed, data was transferred to the University of Pittsburgh through a data transfer agreement.

**Fig 6:**
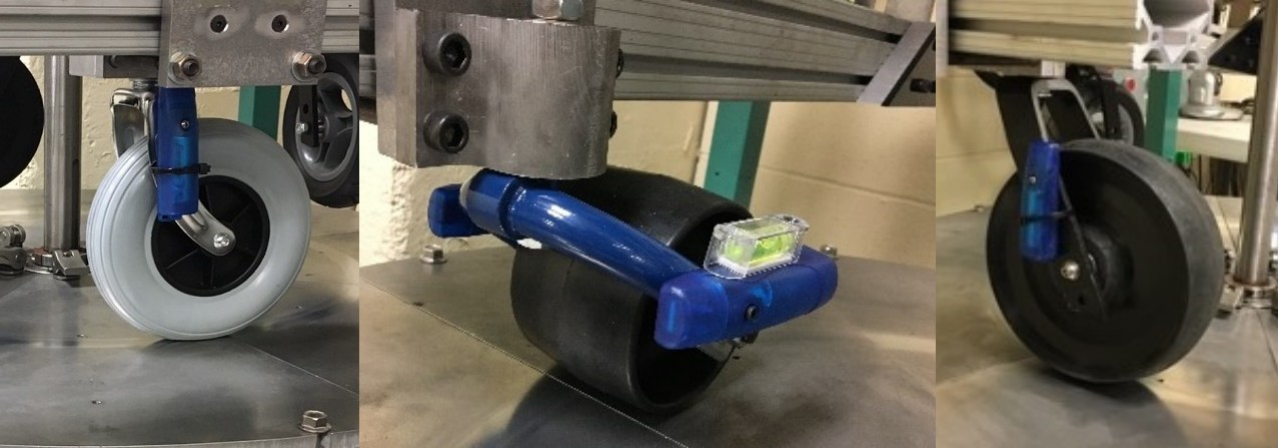
Accelerometers installed on test castors. The Hopehaven castor (left) is a 185mm solid tyre castor that mounts on a plastic castor wheel. The Whirlwind castor (middle) is a 127mm solid tyre castor mounted on the axle bearing similar to the Motivation castor (right) which is a heavy, 216mm solid castor.

Accelerations on the same castor models were recorded on the castor test (Fig 6). The ISO-7176 double drum test standard features obstacles of 6.35mm and 12.7mm shocking wheelchair castor and wheels at 1m/s. For experimentation, multiples of standard obstacle sizes and relevant manual wheelchair speeds were employed. Shock exposure on the test was simulated using obstacles of 6.35mm, 12.7mm, 19.05mm and 25.4mm at four speeds of 0.5m/s, 0.75m/s, 1m/s and 1.25m/s. Typical load on manual wheelchair castors varies between 20-40lbs and standard load for castor testing is 30lbs [19]. For the purpose of this experiment, castors were loaded with least weight– 20lbs as the community data was collected with paediatric participants. Accelerations were collected for one-minute duration. Two castor samples of each model were used to avoid one-off results.

Community accelerations were processed through a bending stress analysis to determine the range of accelerations that cause fatigue. The community and castor test acceleration distributions were matched to determine shock exposure on the castor test and correlated using Chi-squared goodness of fit test.

### Determining corrosion exposure based on outdoor corrosion rates

Corrosion is caused by electrochemical reaction of metals with the environment resulting in oxidation of metals which damages the surface, induces crack formation and degrades material strength.

**Fig 7:**
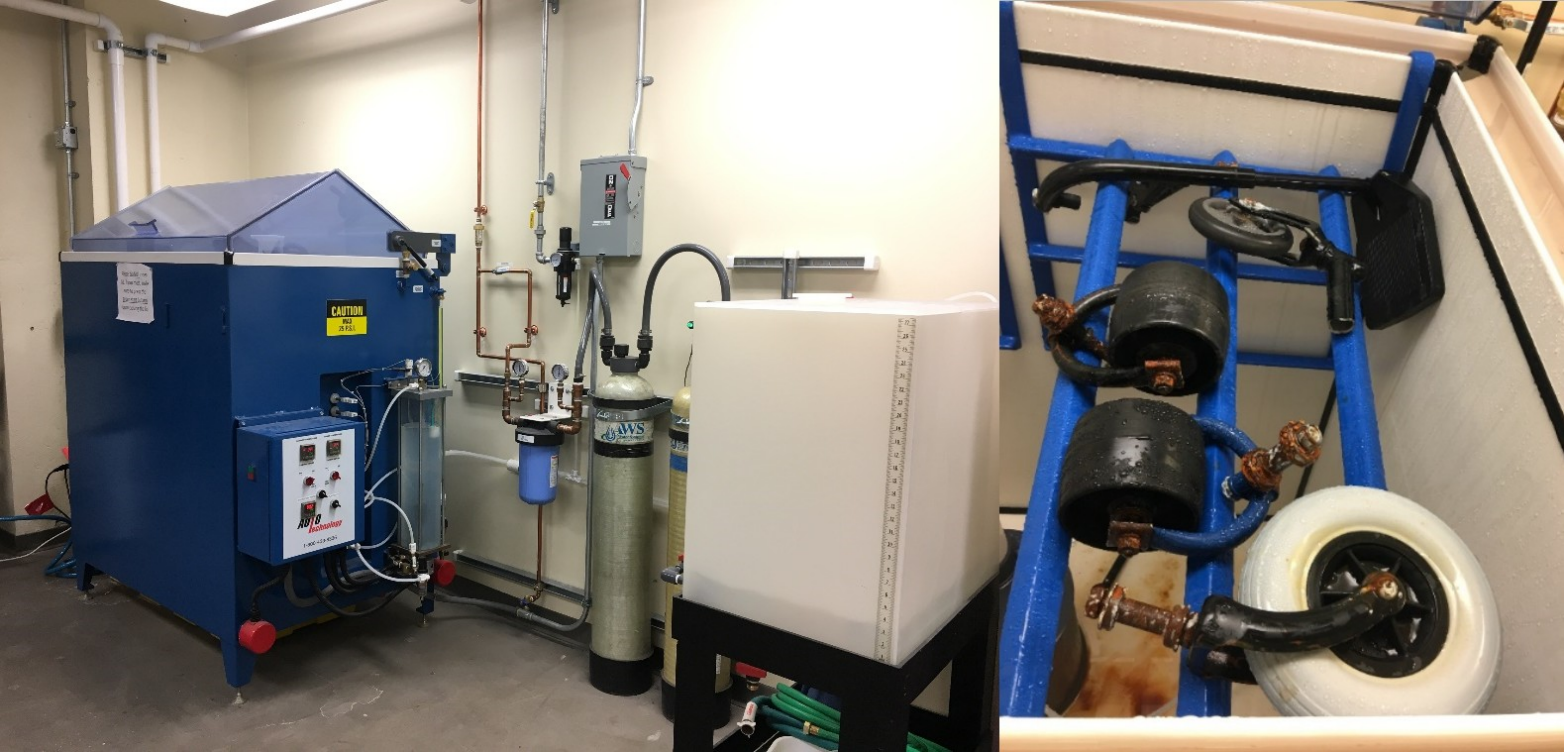
**Salt fog chamber for corrosion testing (left) and corrosion testing of sample parts (right)** [50].

Outdoor steel corrosion rates reported in different parts of the world were collected. Corrosion testing in the laboratory was carried out as per the ASTM B117 standard [48]. The standard recommends a salt fog apparatus (Fig 7) and procedures for corrosion evaluation. An experiment with mass loss test panels was performed to determine the amount of corrosion seen in the salt fog over time. Based on the results, the corrosion rate experienced in the salt fog was calculated using ASTM G1 [49] and correlated with outdoor corrosion rates.

### Determining abrasion exposure based on tyre wear in the community

Tyre abrasion is caused by friction between the tyre and ground during rolling and sliding contact which results in tyre material and tread loss. In this study, abrasion was measured as change in tyre’s outer diameter over a specified time interval.

Failed tyre samples of three wheelchair models–Free Wheelchair Mission (FWM) rubber castor (n = 3), Hopehaven Kids Wheelchair (n = 2) and Motivation Rough Terrain (n = 1) were collected from Kenya for benchmarking abrasion exposure. The period of castor use was recorded in months. The difference between the outer diameters of the used and new castors was measured at three different points on the tyre periphery to compute the yearly wear experienced by the castors.

Simulation of the rough surface on the castor test was done by using sandpaper. Different grit sizes were evaluated with the pattern of obstacles found with shock validation approach. Sandpaper grit was selected based on the one that most closely reproduced the community-based wear rate.

### Validated castor testing

**Fig 8:**
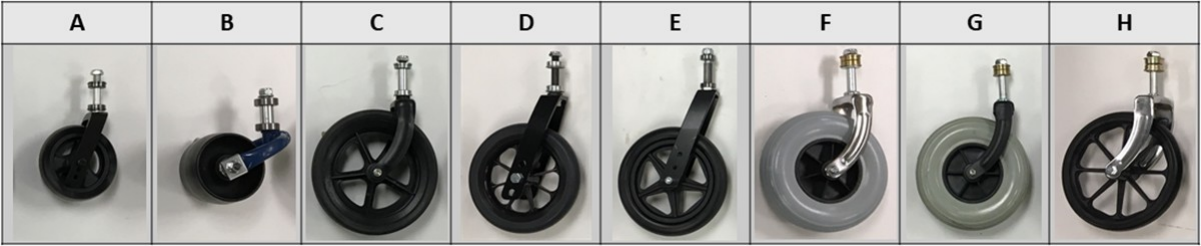
Castor models used for testing.

Eight manual wheelchair castor models (Fig 8) which are representative of castor models used in adverse environments were selected for testing. Models A, B, C, F, and G are used on wheelchairs delivered in LRS. Models D and E are used on standard wheelchairs delivered in RS. Model H represents an institutional wheelchair castor.

Castors were tested under a standard load of 30lbs through four distinct conditions– 1) shock testing; 2) corrosion + shock testing; 3) abrasion + shock testing; and 4) corrosion + abrasion + shock testing. Two samples of each model were tested in each condition, except for models A and F which could not be tested through abrasion conditions because of the limited numbers of samples available.

Samples were tested until the occurrence of a physical failure, which was defined as fracture failure of metallic parts, tyre failure like severe cracking, and/or plastic deformation of castor components. Castor durability was defined as *the number of test cycles completed until a physical failure*. All failure modes observed during the course of testing were recorded as first, second and/or third failure for each sample. Risk of failure mode for causing user injury was assessed based on previously published research [51]. In some cases, testing was terminated following the occurrence of acute failures such as tyre delamination and/or loose axle assembly which could potentially damage the test equipment.

For corrosion testing conditions, samples were exposed to corrosion in the salt fog chamber for 200 hours prior to testing the samples on the castor testing equipment with shocks and/or abrasion. The corrosion exposure time was selected as 200 hours since most castor models fail and need repair and/or replacement within 2 years of use and 100 hours is equivalent to 1 year of corrosion as per the corrosion validation results discussed later [1,5,52].

## Results

Validation of testing factors was conducted to 1 year of community exposure that can be extrapolated as per the required testing protocol duration.

### Shock validation results

#### Correlating shock exposure between community and castor test

**Fig 9:**
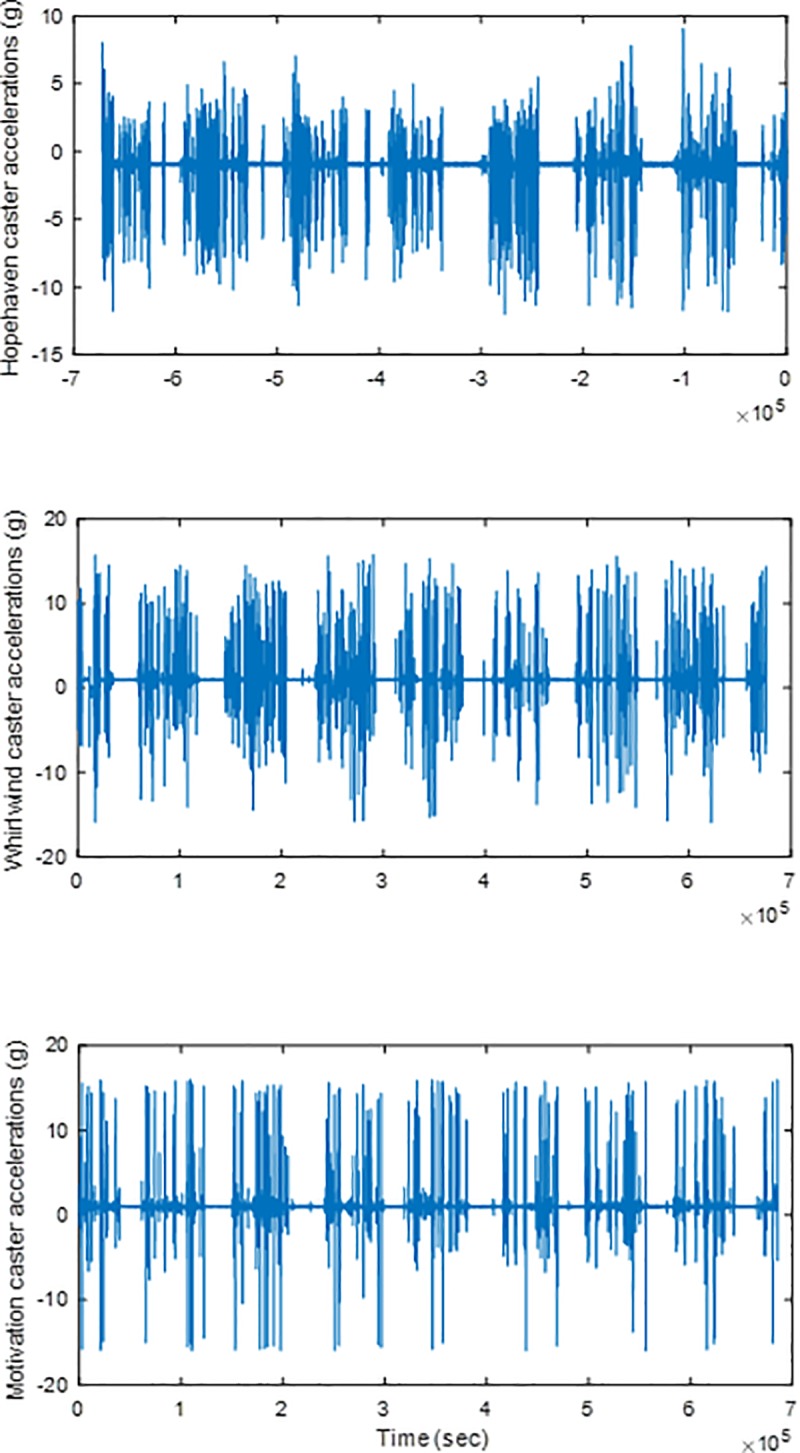
Community accelerations in the vertical direction for three castor models. Out of the two users of Hopehaven wheelchairs, data from the user with higher accelerations is displayed and was selected for analysis to represent the worst-case scenario.

Castor accelerations collected in Kenya for a week are shown in Fig 9. Initial testing and data recording with the Motivation castor showed that higher size obstacles of 25.40mm-31.75mm thickness reproduce community accelerations. This generated shocks that caused the castor test equipment to vibrate significantly. Considering the damage risk, the castor model was omitted from further analysis and testing.

**Fig 10:**
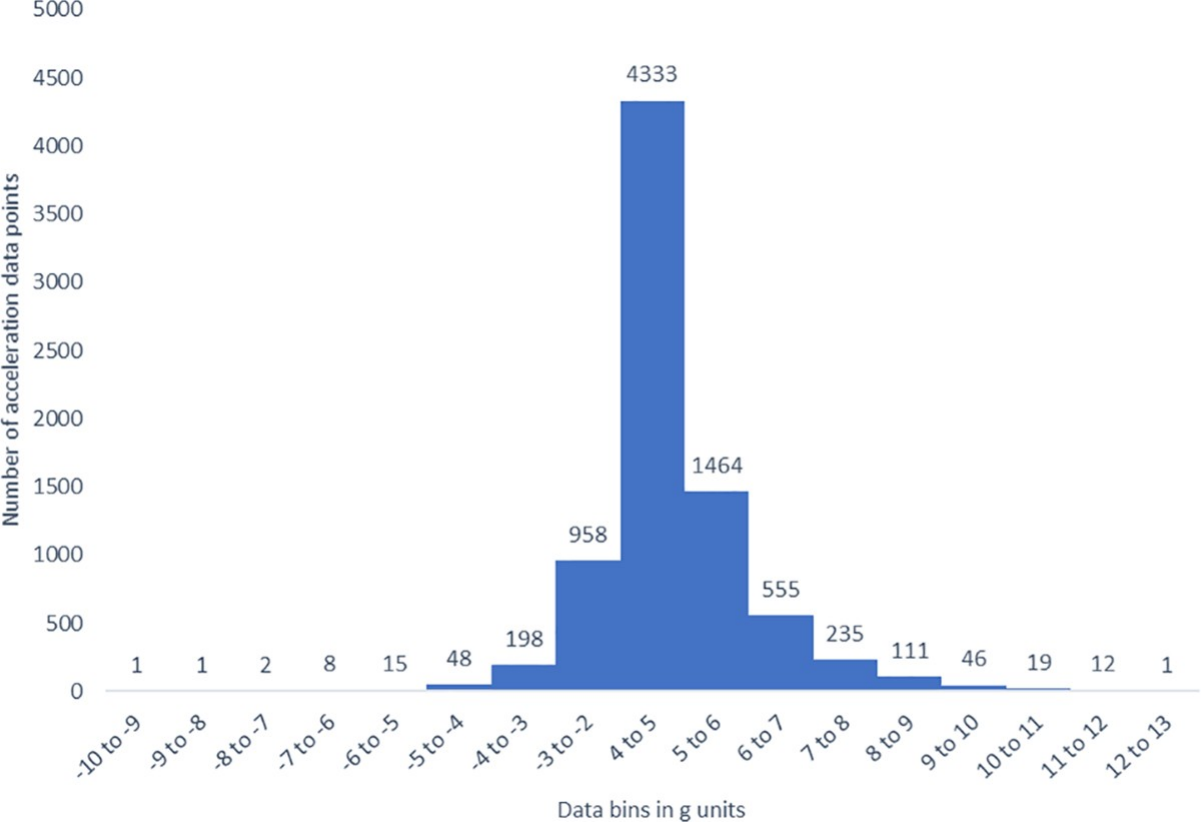
Binning community accelerations recorded on Hopehaven castor. Bins from -2 to 4 g values (mostly surface vibrations) were significantly taller making the high acceleration bins appear flat and hence, are not displayed.

Community accelerations were binned into histograms; for example, accelerations recorded between 8-9g range were binned into one data bin– 8 to 9 g as shown in Fig 10. A curve joining the midpoints of the histogram bins was generated for each castor model.

**Fig 11:**
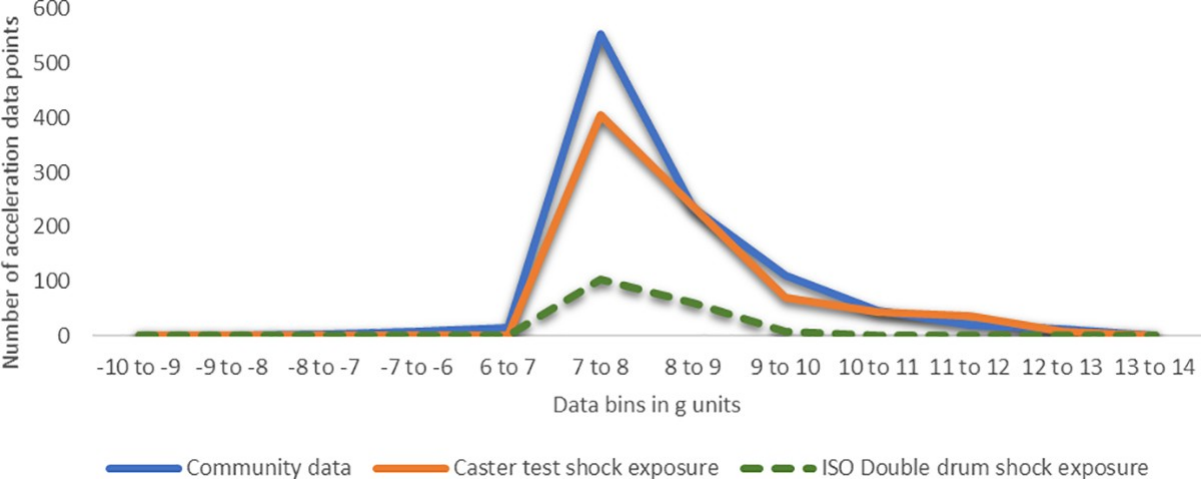
Correlating community and castor test exposures for Hopehaven castor.

**Fig 12:**
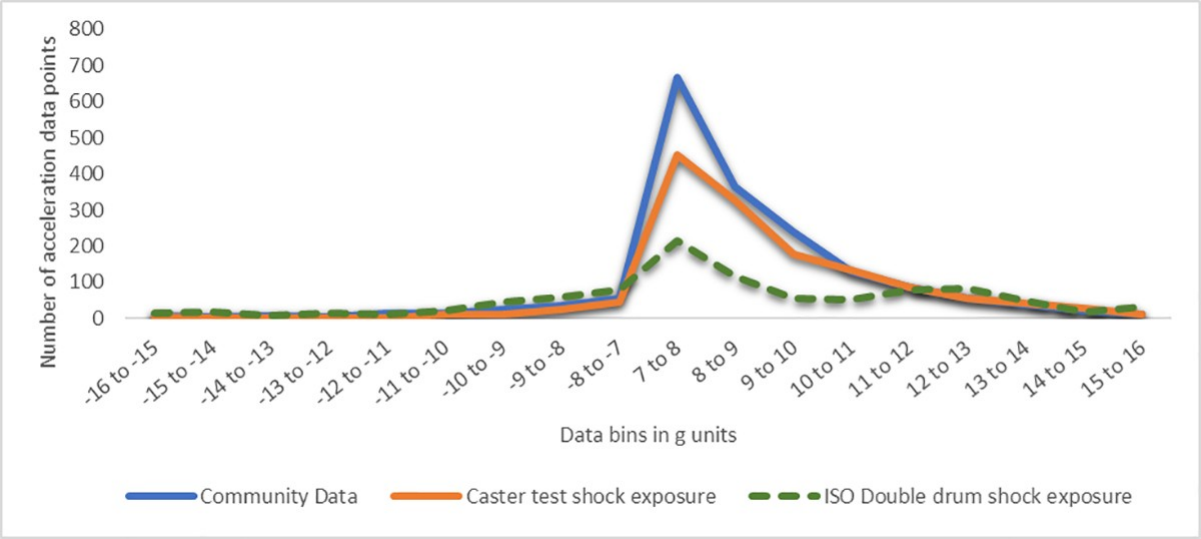
Correlating community and castor test exposures for Whirlwind castor.

According to the stress analysis (see S1 File), accelerations between 6-13g for Hopehaven castor and 7g-16g for Whirlwind castor contributed to fatigue and were considered for correlation between community and castor test. Accelerations recorded on the test with different obstacles and speed settings for one-minute duration were extrapolated and combined suitably to match community acceleration distribution for both models. Figs 11 and 12 show the matched community and castor test acceleration distributions. In these figures, the ISO Double drum exposure is the castor test acceleration distribution prior to this community validation experiment.

Chi-square goodness of fit testing showed that there is a significant difference between the community and validated castor test shock exposures, $\chi_{Hopehaven}^{2}$(11, 24) = 99, p<<0.05, $\chi_{Whirlwind}^{2}$(17, 36) = 128.38, p<<0.05, as well as between community and ISO double drum shock exposures, $\chi_{Hopehaven}^{2}$(11, 24) = 695.78, p<<0.05, $\chi_{Whirlwind}^{2}$(17, 36) = 856.47, p<<0.05. Following validation, the gaps between community and castor test acceleration distributions were smaller, suggesting the castor test system provides an exposure more similar to the community than the ISO test. Higher magnitude accelerations greater than 10g that contribute greater to fatigue stress in the castor’s stem bolt were matched appropriately with shock validation.

#### Shock testing protocol

Shock validation provided the duty cycle or number of test cycles to simulate community shocks experienced in a year and yielded two separate shock testing protocols based on castor size (Table 1). In each protocol, shock exposure was divided into low and high magnitudes as obstacles of different heights were included. For castors, less than 6 inches in diameter, the low and high magnitude shocks can be simulated at the same time on the turntable unlike the castors with a larger diameter, which requires sequential testing. Castors reverse their direction of travel for approximately 10% of the total travel which was simulated by rotating the turntable in reverse every 900 forward cycles [53].

**Table 1:** Validated testing protocols for shock testing.

Exposure	Test cycles for one-year exposure	Bump Height (mm)	Number of obstacles	Speed	Direction of turntable rotation (test cycles)
Shock testing protocol I: Castors less than 150mm in diameter (e.g. Whirlwind model)					
Low-magnitude	3000	6.35	n = 2	1m/s	Forward (2700) Reverse (300)
High-magnitude		12.7	n = 1	1m/s	
Shock testing protocol II: Castors greater than or equal to 150mm in diameter (e.g. Hopehaven model)					
Low-magnitude	4500	12.7	n = 2	1m/s	Forward (4050) Reverse (450)
High-magnitude	1500	19.05	n = 1	1m/s	Forward (1350) Reverse (150)

### Corrosion validation results

#### Collecting outdoor corrosion rates

Table 2 shows the published corrosion rates for different countries [54–62]. Rates for carbon steel were selected since the mass loss test panels used for validation were made of SAE 1008 carbon steel.

**Table 2:** Carbon steel corrosion rates seen in different countries.

Country	Corrosion rate in mm/year
China	0.1–0.9
India	0.04–1.60
Saudi Arabia	0.0023–0.536
Mexico	0.01–0.3
Colombia	0.01–0.17
Canary Islands, Spain	0.004–0.263
Australia	0.35–0.42
South Africa	0.05–0.26
Japan	0.08–0.89
United States	0.01–1.07
South Africa	2.19
Panama	0.99
Kenya	0.05

#### Corrosion testing in the salt fog chamber

**Fig 13:**
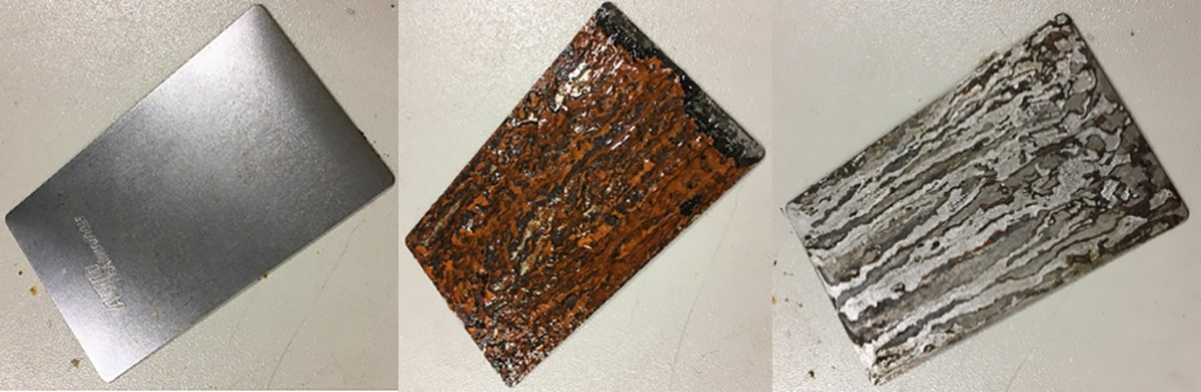
Mass loss test panel before corrosion (left), corroded panel after 100hrs of salt fog exposure (centre) and cleaned panel before weighing (right).

Three test panels were cleaned and placed in the salt fog chamber for constant fog exposure at 48°C with high relative humidity (about 97%). Weight loss on test panels was evaluated on a scale at 100, 200 and 300 hours of exposure. Prior to weighing, panels were cleaned, and all rust was scraped from the panels (see Fig 13) surface using a scrubber as specified in ASTM G1 and ASTM B117.

#### Corrosion testing protocol

Mass loss seen with the three panels after every 100 hours of exposure ranged from 1.33–1.50 grams. Accordingly, the corrosion rate average for the three panels comes to 1.5mm/year. Comparing this result with the outdoor corrosion rates in Table 3, 100 hours of salt fog exposure can simulate corrosion equivalent to 1 year of outdoor corrosion.

**Table 3:** Outdoor abrasion rates.

Model	Period of use (months)	Number of castors	Tyre material loss per year (mm)
FWM rubber tyre	14.67 ± 4.62	n = 3	10.03 ± 0.86
Hopehaven		n = 2	1.40 ± 0.08
Motivation	8.00	n = 1	0.95

### Abrasion validation results

#### Tyre wear observed in the community

Table 3 shows the tyre wear experienced by different castor models.

#### Conducting abrasion simulation on castor test

**Fig 14:**
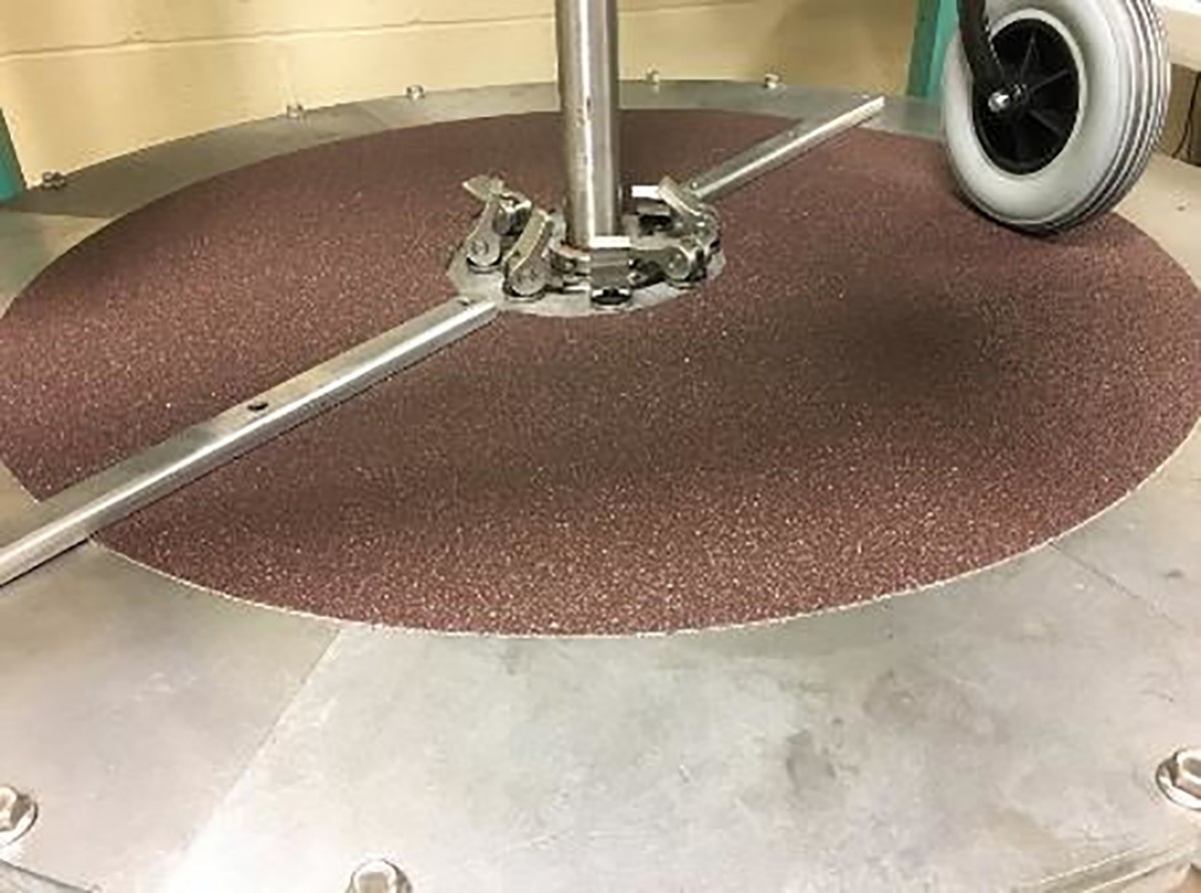
36-grit sanding disc attached to the turntable to simulate abrasion.

Sandpapers of 20 and 36 grit sizes were evaluated individually by attaching them to the turntable to simulate rough surface. Obstacles were bolted through the sandpaper and the pattern was based on validated shock testing protocol for castors greater than 150mm in diameter. Fig 14 shows the test setup with the 36-grit, 0.76m wide sanding disc. Two new Hopehaven castor samples were tested. Reduction in tyre thickness was calculated following shock exposure corresponding to one year of outdoor exposure. The tested models experienced wear of 1.84 ± 0.7mm with 36-grit sandpaper compared to 0.51 ± 0.03mm with the 20-grit sandpaper. The 20-grit sandpaper had less number of grits per square area with a high grit profile which resulted in less material removal. Comparing the abrasion rate with that experienced in the community (Table 3), the 36-grit sandpaper was found suitable for abrasion testing.

### Validated castor testing results

**Fig 15:**
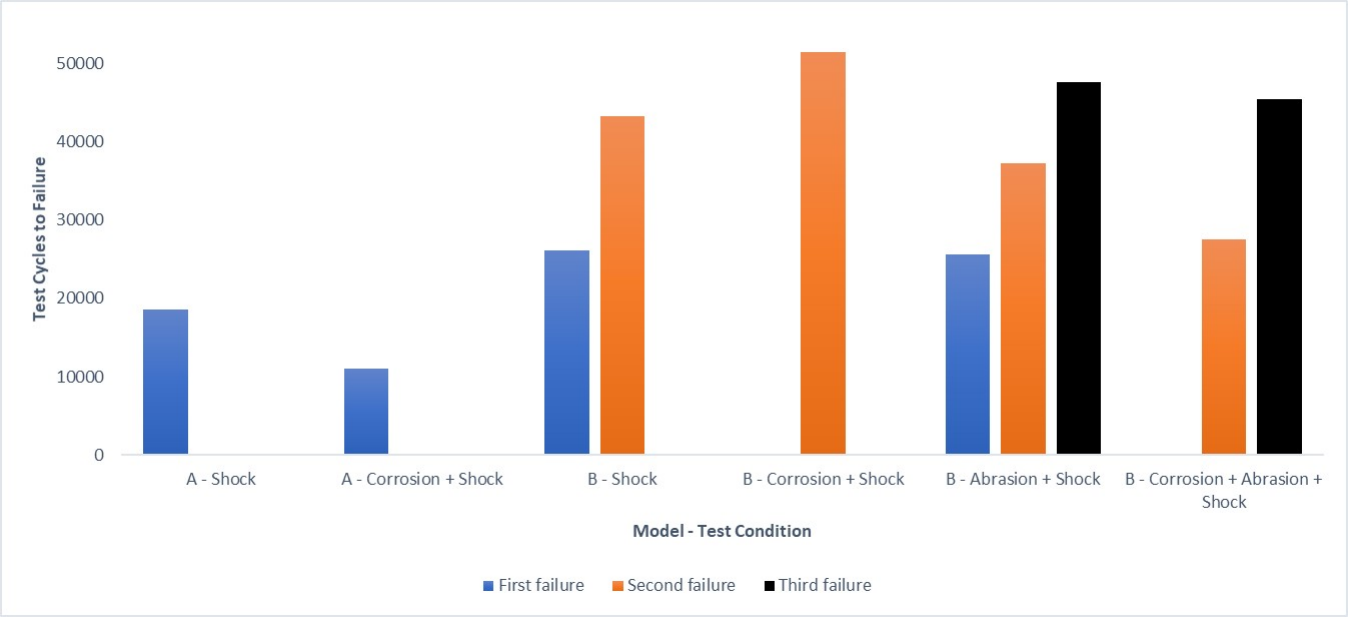
Test cycles to failure for small castors (less than 150mm in diameter). The number of failures for the two samples in each condition varied across models and test conditions. Corrosion tested samples of B suffered obstruction to rolling due to corrosion exposure and hence, no testing cycle is noted for their first failure.

**Fig 16:**
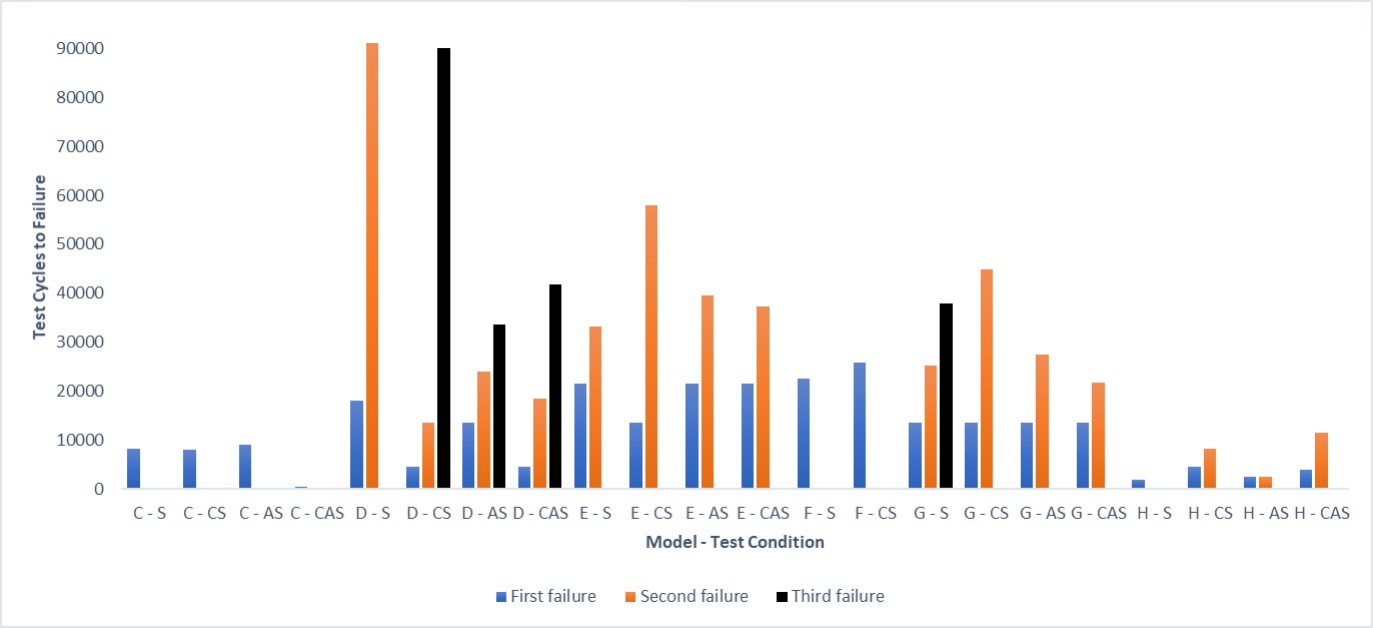
Test cycles to failure for larger castors (greater than or equal to than 150mm in diameter). The number of failures for the two samples in each condition varied across models and test conditions. Abbreviations: S, Shock; CS, Corrosion + Shock; AS, Abrasion + Shock; CAS, Corrosion + Abrasion + Shock.

**Fig 17:**
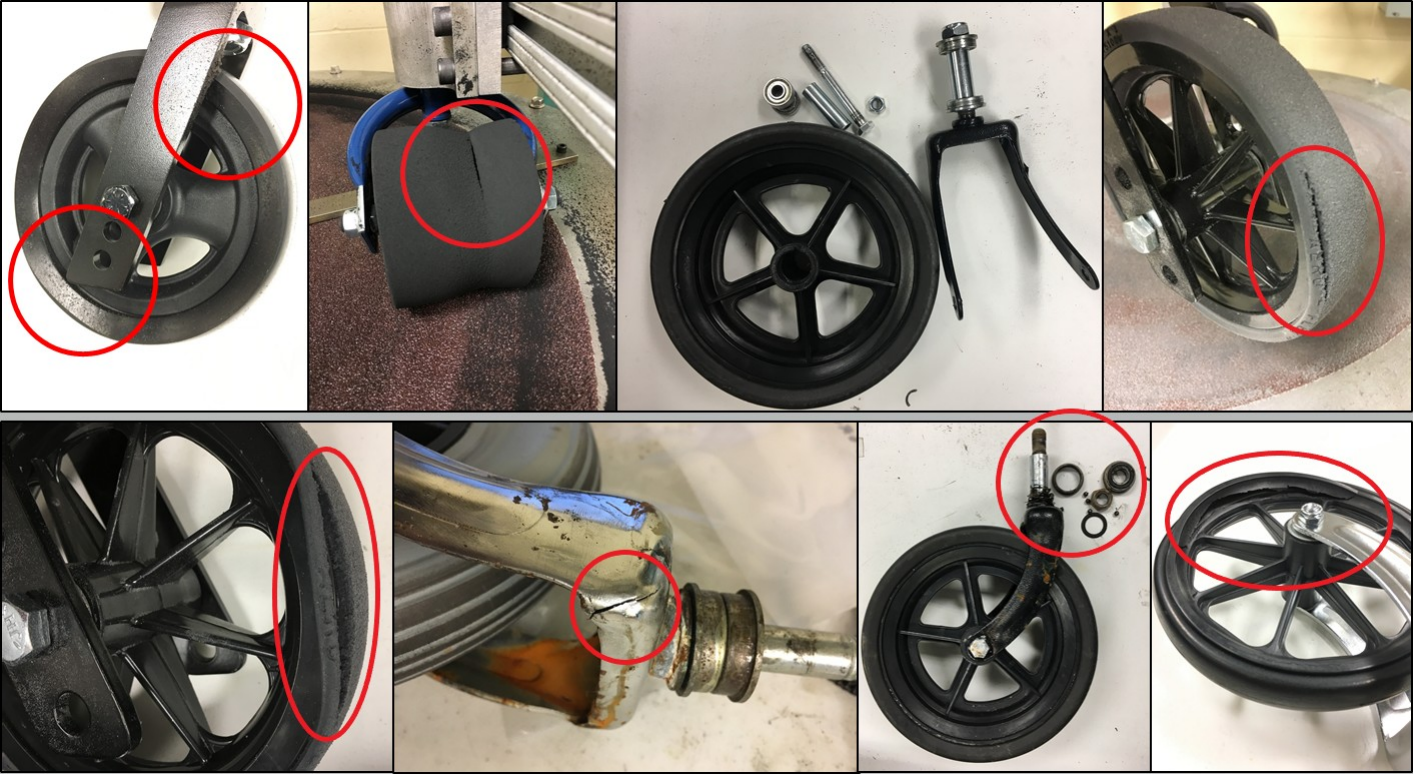
Model A–Delaminated tyre brushing against the fork (top-left), Model B–Tyre cracking (top-left-middle), Model C–Axle hub assembly fell apart (top-right-middle), Model D–Tyre Cracking (top-right), Model E–Tyre Cracking (bottom-left), Model F–Fork fracture (bottom-left-middle), Model G–Stem bearing fracture (bottom-right-middle) and Model H–Wheel Fracture (bottom-right).

Castor models A and B were less than 150mm in diameter and were tested with shock testing protocol I. The other models were tested with shock testing protocol II. As per the results, the castor durability and failure modes of seven out of eight models (87.5%) were affected by corrosion and abrasion. Figs 15 and 16 show the test cycles and Table 4 shows the castor failure modes. Stem bearings from corroded samples of B, D and G locked randomly during testing which led to dampening of the castor’s lateral swivel. This phenomenon was associated with four corroded samples that lasted more test cycles before failure than the non-corroded samples. Among tested models, models C and H failed quite early across all four testing conditions compared to others. Fig 17 shows the example photographs of failures.

**Table 4:** Castor failure modes.

Model—Test Condition	First failure mode	Second failure mode	Third failure mode
A—S	Tyre delamination		
A—CS	Tyre delamination, Stem bearing corrosion		
B—S	Loose stem bearings	Fork crack*	
B—CS	Severe rolling obstruction	Fork crack*	
B—AS	Tyre cracking** (1), Loose stem bearings	Fork crack* (1), Tyre cracking** (2)	
B—CAS	Severe rolling obstruction	Loose stem bearings	Tyre cracking**
C—S	Axle hub failure* (1), Stem bolt fracture* (2)		
C—CS	Stem bearing fracture (1), Axle hub failure* (2), All bearings corroded		
C—AS	Axle hub failure* (1), Fork fracture* (2), Stem bolt bent* (2), Tyre tread worn-out (2)		
C—CAS	Wheel fracture*, Fork corrosion		
D—S	Loose axle bearings	Axle hub failure*	
D—CS	Loose axle bearings	Loose, noisy stem bearings	Axle hub failure*
D—AS	Loose axle bearings	Tyre delamination**	Tyre cracking **
D—CAS	Loose axle bearings, Stem bearing corrosion	Loose, noisy stem bearings	Tyre cracking **
E—S	Loose stem bearings	Stem bolt fracture*	
E—CS	Locked, corroded stem bearings	Stem bolt fracture*, Bent bolt**	
E—AS	Loose stem bearings	Tyre cracking** (1), Stem bolt fracture* (2)	
E—CAS	Loose, corroded stem bearings	Stem bolt fracture*, Tyre cracking**	
F—S	Bent fork*		
F—CS	Fork fracture**		
G—S	Loose and locked stem bearings	Axle hub loose (1), Stem bearing fracture (2)	Stem bearing fracture (1)
G—CS	Loose and locked stem bearings	Stem bearing fracture	
G—AS	Loose and locked stem bearings	Stem bearing fracture, Bent bolt** (2)	
G—CAS	Loose and locked stem bearings	Fork fracture** (1), Stem bearing fracture (2), Bent bolt** (2)	
H—S	Wheel fracture*		
H—CS	Tyre delamination	Fork fracture**	
H—AS	Tyre delamination	Stem bolt bent**	
H—CAS	Tyre delamination	Fork fracture**	

Failure modes are numbered according to sample number in case both the samples in the test condition experienced different failure modes. Testing was continued for models D and H following tyre delamination as the failures did not pose a risk for damaging the equipment as model A.

Abbreviations for test conditions: S, Shock; CS, Corrosion + Shock; AS, Abrasion + Shock; CAS, Corrosion + Abrasion + Shock

* Failures mode associated with high risk for user injury

** Failure mode different than baseline shock condition due to corrosion/abrasion exposure

## Discussion

Wheelchair parts, especially castors, experience frequent failures in the community [1–3,5,19,21,63]. These failures pose a risk for additional wheelchair failures and user consequences like being injured, stranded and missing work and school. To prevent failures, design and quality improvements are needed, and laboratory-based castor quality testing can provide such information. A castor testing system was guided and motivated by the ISWP-SWG, and allows the castor systems to be exposed to a range of environmental factors, including shock, abrasion, corrosion and debris infiltration [41]. The goals of this study were to develop a testing protocol that was based on community exposures to shock, abrasion and corrosion and evaluate the effect of environmental factors of corrosion and abrasion on the castor durability and failure mode.

Table 5 summarizes the results of castor testing with environmental factors.

**Table 5:** Summary of testing results.

Castor Model	Impact to Durability		Impact to Failure Mode	
	Abrasion	Corrosion	Abrasion	Corrosion
A	Not tested	Reduced Durability	Not tested	Same
B	Almost same durability	Reduced Durability	Changed	Changed
C	Unclear	Unclear	Unclear	Unclear
D	Reduced Durability	Reduced Durability	Changed	Same
E	Same durability	Reduced Durability	Changed	Same
F	Not tested	Almost same durability	Not tested	Changed
G	Reduced Durability	Same durability	Changed	Same
H	Unclear	Unclear	Unclear	Changed

### Effect of corrosion and abrasion factors on castor durability

Corrosion affected the durability of four out of the eight tested models causing them to loosen, lock or become sticky. The corroded stem and axle bearings of models B, D, and E failed first and saw a reduction from 37% to 100% in test cycles to failure compared to shock only condition. Corrosion had a significant influence on the sleeve axle bearings of model B which experienced severe resistance following corrosion testing. In addition to bearings, corrosion affected tyres. Exposure to hot and humid condition in the salt fog caused model A tyres to loosen earlier compared to shock only testing condition and begin rolling-off the wheel. This failure mode seems to occur with some polyurethane tyres; it was witnessed with another model during preliminary castor testing [19]. A reduction of nearly 40% in durability was seen with model A samples due to corrosion.

An unexpected result from corrosion testing was the random locking or stiffening of some stem bearings. Shocks on the castor test were not enough to break loose the failed bearings and testing was allowed to continue until the occurrence of a physical failure. The stiff or locked bearings experienced less swivelling, which was associated with increased durability for a few samples. These stiff bearings may fracture if impact was included as a testing factor in the protocol simulating the effect of a kerb drop similar to the wheelchair kerb-drop testing in ISO 7176 section 8. This will be investigated in future work.

Abrasion impacted durability of two models–D and G; tyres were found to wear out, crack and etch on the sides with sandpaper testing. In the majority of cases, tyre failures were second or third failures following bearing failures. With the added influence of abrasion, there was a significant reduction in the durability of model D samples by 63%, p<0.05. Model G saw a reduction of 13% however, it had a change in failure mode which is discussed later.

Across all models, with a few exceptions, corrosion and abrasion affected castor durability. This finding is consistent with the anecdotal feedback from manufacturers noted in the introduction that the environmental factors impact the durability of castor parts like bearings and tyres in the community. Few exceptions were found when the number of test cycles for models of B, D, E, F and H was slightly higher with added environmental testing factor (s). It should be noted that such variations in results within model groupings are commonly found in wheelchair testing studies that subject wheelchairs to a ISO-7176 standard exposure [64–67].

Castor models with loose engineering tolerances between castor parts, harder tyres and bearings with lower load capacities failed early compared to their time to failure documented in the previous castor testing study [19]. High durometer tyres on these castors are unable to absorb shocks which affect the rest of the assembly causing fasteners to loosen up, and bearings and bolts to fracture. Premature failures made it difficult to determine the impact of environmental factors on castor durability. On the other hand, consistent results were found with models with higher part mating tolerances and bearing load capacities. Samples of models A, B, D and E lasted an approximately equal number of test cycles to failure and experienced similar failure modes within each of the four testing conditions (exceptions with samples of B and E in shock + abrasion condition) which demonstrates the strong internal validity of the testing protocol.

### Effect of corrosion and abrasion factors on failure modes

Failure modes were different across six models when tested with the environmental factors of corrosion and abrasion. For instance, failure modes for four models–B, D, E and G were altered by the presence of abrasion, and three models–B, F and H in the presence of corrosion. Two-thirds of the altered failure modes–bent bolt and fork fracture, are associated with increased risk of causing user injuries based on a castor failure checklist [68]. The forks fractured at corroded hotspots.

Model C experienced mixed failure modes which made it difficult to determine whether environmental factors had any effect on the catastrophic failure modes. Lack of part mating tolerances, inappropriate materials, lower load capacity of bearings and/or unsuitable coatings may have contributed to these failures.

## Study implications

Corrosion and abrasion significantly impacted the castor durability and failure modes of tested models. The results of this study support the inclusion of corrosion and abrasion in the castor testing protocol as well as wheelchair durability testing (ISO 7176), which currently does not require these conditions.

Screening of castor designs that are inappropriate for use in adverse environments has been the prime objective behind developing the castor testing equipment and the protocol. The shock testing condition alone, as observed in both castor testing studies, can expose weak links in the castor designs such as lower load capacity of bearings, hard tyres and loose tolerances between parts which can lead to catastrophic failures. With the inclusion of corrosion, the testing protocol is now able to identify bearings that corrode and lose function which makes them unsuitable for hot and humid climates. The abrasion condition makes tyre designs with less tyre depth and minimal abrasion resistance wear out during testing. Thus, including the environmental factors of corrosion and abrasion has improved the testing protocol’s capability for screening inappropriate designs.

Castor testing results found in this study were immediately useful in two ways. First, manufacturers and providers were informed about the results and corresponding design improvements. This feedback was welcomed and additional testing following design improvements are planned. Second, castor design and selection guidelines based on these results were compiled to benefit designers, technicians and manufacturers who plan to design and develop castors for use in adverse environments [69]. Providers can use the guidelines for selecting castor designs with regards to the context of use.

### Limitations

The testing protocol was developed based on samples of only a few castors in the community which is a primary limitation of this study. The shock data collected in Kenya did not include information such as castor load, condition, maintenance status and age. Also, information about users’ health, weight and activity levels was unavailable. The shock data was collected from the paediatric population only which is a limitation. As the castor load varies typically from 20-40lbs, the authors used 20lbs as the load for validation purposes which is not the same as the standard castor load of 30lbs [19]. Although a limitation, our approach to developing standards testing protocols from community data is relatively unique to the standards development process for wheelchairs, which has historically been consensus-based and not evidence-based.

Accelerations provide partial evidence for fatigue analysis and shock validation. The physical effect of instantaneous accelerations can only be evaluated by measuring strains and forces which was not done as a part of this study. There were limitations related to the accelerometer data. Shocks and impacts above ±16g which significantly contribute to fatigue could not be recorded in the community and on the castor test due to limitations with the sensor specifications.

Finally, a relatively small sample size of castors was available for testing. Due to uncontrolled variables, such as manufacturing variability, testing more samples would help strengthen the results. The number of castor samples of each model selected for validation testing was dependent on the number of samples that were supplied by manufacturers.

### Future work

The authors plan to conduct further validation studies focusing on collecting more extensive community data on shocks, impacts and environmental factors. The authors plan to investigate the effect of large impacts as a testing factor in the protocol which simulates the effect of a kerb drop similar to the wheelchair kerb-drop testing in ISO 7176 section 8. Another step in the protocol development is correlating lab-based castor failure modes with community failures to evaluate the external validity of the protocol. To that end, the authors have initiated the community data collection on failure modes using the validated C-FIT tool [51].

The ISWP-SWG has developed a design considerations document to guide wheelchair manufacturers, designers, providers and researchers on wheelchair design and development for use in adverse environments [69]. The authors plan to incorporate the castor design guidelines in the design considerations document. For dissemination purposes, the authors plan to publish the castor testing protocol as a technical specification which will be an add-on to the current ISO 7176 wheelchair standards. To support the global implementation of castor testing protocol, ISWP-SWG will be publishing informational materials and drawings of the castor testing system.

## Conclusions

Wheelchair castors experience frequent failures in the adverse environments experienced in LRS and rural areas of RS. To prevent failures and subsequent user consequences, the WHO Guidelines recommend testing products based on outdoor exposure in adverse environments which can inform design improvements. The ISWP-SWG developed a castor testing system and this study presented the development and evaluation of the castor testing protocol. Community-matching exposures of shock, corrosion and abrasion were added to the testing protocol. Testing castor models through the new validated protocol showed that the environmental factors of corrosion and abrasion affect castor durability and failures modes which supports their inclusion in product testing. Further validation is required to improve the castor failure prediction accuracy of the testing protocol.

## Supporting information

S1 File Supporting information file. (DOCX)

S1 Fig Stem bolt fracture failure. (TIFF)

## References

[1] Rispin K, Riseling K, Wee J. A longitudinal study assessing the maintenance condition of cadres of four types of wheelchairs provided in low-resource areas. Disabil Rehabil Assist Technol. England; 2018 2;13(2):146–56. 10.1080/17483107.2017.1299805 28326868

[2] Toro M, Worobey L, Boninger ML, Cooper RA, Pearlman J. Type and Frequency of Reported Wheelchair Repairs and Related Adverse Consequences Among People With Spinal Cord Injury. Arch Phys Med Rehabil. W.B. Saunders; 2016 10;97(10):1753–60. 10.1016/j.apmr.2016.03.032 27153763

[3] Saha R, Dey A, Hatoj M, Podder S. Study of wheelchair operations in rural areas covered under the District Rehabilitation Centre (DRC) scheme. Indian J Disabil Rehabil. 1990;(Jul-Dec):57–87.

[4] Armstrong W, Reisinger KD, Smith WK. Evaluation of CIR-whirlwind wheelchair and service provision in Afghanistan. Disabil Rehabil [Internet]. 2007 1 [cited 2015 Mar 9];29(11–12):935–48. Available from: http://www.ncbi.nlm.nih.gov/pubmed/17577728 10.1080/09638280701240615 17577728

[5] Reese N, Rispin K. Assessing Wheelchair Breakdowns In Kenya To Inform Wheelchair Test Standards For Low-Resource Settings. In: RESNA Annual Conference—2015. 2015.

[6] Toro ML, Garcia Y, Ojeda AM, Dausey DJ, Pearlman J. Quantitative Exploratory Evaluation of the Frequency, Causes and Consequences of Rehabilitation Wheelchair Breakdowns delivered at a Paediatric Clinic in Mexico. Disabil CBR Incl Dev [Internet]. 2012 12 8 [cited 2015 Jul 23];23(3):48–64. Available from: http://dcidj.org/article/view/167

[7] Mukherjee G, Samanta A. Wheelchair charity: a useless benevolence in community-based rehabilitation. Disabil Rehabil [Internet]. Informa UK Ltd UK; 2005 5 20 [cited 2015 Mar 2];27(10):591–6. Available from: http://www.ncbi.nlm.nih.gov/pubmed/16019868 10.1080/09638280400018387 16019868

[8] Worobey L, Oyster M, Nemunaitis G, Cooper R, Boninger ML. Increases in wheelchair breakdowns, repairs, and adverse consequences for people with traumatic spinal cord injury. Am J Phys Med Rehabil [Internet]. 2012 6 [cited 2015 Jul 28];91(6):463–9. Available from: http://www.ncbi.nlm.nih.gov/pubmed/22549473 10.1097/PHM.0b013e31825ab5ec 22549473 PMC4886332

[9] Mair C. Applied internet-of things technology in the management of wheelchair maintenance at NHS WestMARC: A retrospective. In: European Seating Symposium. 2018.

[10] Worobey L, Oyster M, Pearlman J, Gebrosky B, Boninger ML. Differences between manufacturers in reported power wheelchair repairs and adverse consequences among people with spinal cord injury. Arch Phys Med Rehabil [Internet]. 2014 4 [cited 2015 Jul 28];95(4):597–603. Available from: http://www.ncbi.nlm.nih.gov/pubmed/24361786 10.1016/j.apmr.2013.11.022 24361786

[11] Hogaboom NS, Worobey LA, Houlihan B V, Heinemann AW, Boninger ML. Wheelchair breakdowns are associated with pain, pressure injuries, rehospitalization, and self-perceived health in full-time wheelchair users with spinal cord injury. Arch Phys Med Rehabil. 2018; 10.1016/j.apmr.2018.04.002 29698640

[12] Gaal RP, Rebholtz N, Hotchkiss RD, Pfaelzer PF. Wheelchair rider injuries: causes and consequences for wheelchair design and selection. J Rehabil Res Dev [Internet]. 1997 1 [cited 2015 Jul 26];34(1):58–71. Available from: http://www.ncbi.nlm.nih.gov/pubmed/9021626 9021626

[13] Kirby RL, Ackroyd-Stolarz SA, Brown MG, Kirkland SA, MacLeod DA. Wheelchair-related accidents caused by tips and falls among noninstitutionalized users of manually propelled wheelchairs in Nova Scotia. Am J Phys Med Rehabil. United States; 1994;73(5):319–30. 10.1097/00002060-199409000-00004 7917161

[14] Kim J, Mulholland S. Seating/wheelchair technology in the developing world: need for a closer look. Vol. 11, Technology and Disability. IOS Press; 1999 p. 21–7.

[15] Borg J, Lindström A, Larsson S. Assistive technology in developing countries: a review from the perspective of the Convention on the Rights of Persons with Disabilities. Prosthet Orthot Int. 2011 3;35(1):20–9. 10.1177/0309364610389351 21515886

[16] Pearlman J, Cooper RA, Krizack M, Lindsley A, Wu Y, Reisinger KD, et al Lower-limb prostheses and wheelchairs in low-income countries. IEEE Eng Med Biol Mag [Internet]. 2008 1 [cited 2015 Jul 26];27(2):12–22. Available from: http://www.ncbi.nlm.nih.gov/pubmed/18463017 10.1109/EMB.2007.907372 18463017

[17] Hotchkiss R. Independence through Mobility: A guide through the manufacture of the ATI-Hotchkiss Wheelchair. 1985.

[18] Toro ML, Eke C, Pearlman J. The impact of the World Health Organization 8-steps in wheelchair service provision in wheelchair users in a less resourced setting: a cohort study in Indonesia. BMC Health Serv Res. 2016;16(1):26. 26801984 10.1186/s12913-016-1268-y PMC4722611

[19] Mhatre A, Ott J, Pearlman J. Development of wheelchair caster testing equipment and preliminary testing of caster models. African J Disabil. AOSIS; 2017 9;6:358. 10.4102/ajod.v6i0.358 PMC5645576 29062762

[20] Gaal RP, Rebholtz N, Hotchkiss RD, Pfaelzer PF. Wheelchair rider injuries: causes and consequences for wheelchair design and selection. J Rehabil Res Dev. 1997 1;34(1):58–71. 9021626

[21] Toro ML, Garcia Y, Ojeda AM, Dausey DJ, Pearlman J. Quantitative Exploratory Evaluation of the Frequency, Causes and Consequences of Rehabilitation Wheelchair Breakdowns delivered at a Paediatric Clinic in Mexico. Disabil CBR Incl Dev. 2012 12;23(3):48–64.

[22] Toro Hernandez ML. Development, Implementation, and Dissemination of a Wheelchair Maintenance Training Program. University of Pittsburgh; 2016.

[23] Cooper RA, Robertson RN, Lawrence B, Heil T, Albright SJ, VanSickle DP, et al Life-cycle analysis of depot versus rehabilitation manual wheelchairs. J Rehabil Res Dev [Internet]. 1996 2 [cited 2015 May 13];33(1):45–55. Available from: http://www.ncbi.nlm.nih.gov/pubmed/8868417 8868417

[24] Cooper RA, Gonzalez J, Lawrence B, Renschler A, Boninger ML, VanSickle DP. Performance of selected lightweight wheelchairs on ANSI/RESNA tests. American National Standards Institute-Rehabilitation Engineering and Assistive Technology Society of North America. Arch Phys Med Rehabil [Internet]. 1997 10 [cited 2015 Feb 18];78(10):1138–44. Available from: http://www.ncbi.nlm.nih.gov/pubmed/9339166 10.1016/s0003-9993(97)90141-6 9339166

[25] Cooper RA, Boninger ML, Rentschler A. Evaluation of selected ultralight manual wheelchairs using ANSI/RESNA standards. Arch Phys Med Rehabil [Internet]. 1999 4 [cited 2015 Feb 18];80(4):462–7. Available from: http://www.ncbi.nlm.nih.gov/pubmed/10206612 10.1016/s0003-9993(99)90287-3 10206612

[26] Cooper R, Stewart K, VanSickle D, Albright S, Robertson R, Flannery M, et al. Manual Wheelchair ISO-ANSI/RESNA Fatigue Testing Experience. In: Proceedings of the RESNA ‘94 Annual Conference. Nashville, TN; 1994. p. 324–6.

[27] Fitzgerald SG, Yoest LM, Cooper RA. Comparison of Laboratory and Actual Fatigue Life for Three Types of Manual Wheelchairs. In: Proceedings of the RESNA 2001 Annual Conference. Reno, NV; 2001 p. 352–4.

[28] Gebrosky B, Pearlman J, Cooper RA, Cooper R, Kelleher A. Evaluation of lightweight wheelchairs using ANSI/RESNA testing standards. J Rehabil Res Dev [Internet]. Rehabilitation Research and Development Service; 2013 1 [cited 2015 Feb 20];50(10):1373–89. Available from: http://www.scopus.com/inward/record.url?eid=2-s2.0-84896344453&partnerID=tZOtx3y1 10.1682/JRRD.2012.08.0155 24699973

[29] Liu H, Cooper RRA, Pearlman J, Cooper RRA, Connor S, Boninger ML, et al Evaluation of titanium ultralight manual wheelchairs using ANSI/ RESNA standards. Arch Phys Med Rehabil [Internet]. 1999 1 [cited 2015 Feb 18];45(4):1251–67. Available from: http://www.ncbi.nlm.nih.gov/pubmed/10206612 19319751

[30] Liu H, Pearlman J, Cooper R, Hong E, Wang H, Salatin B, et al Evaluation of aluminum ultralight rigid wheelchairs versus other ultralight wheelchairs using ANSI/RESNA standards. J Rehabil Res Dev [Internet]. 2010 1 [cited 2015 Feb 18];47(5):441–55. Available from: http://www.ncbi.nlm.nih.gov/pubmed/20803388 10.1682/jrrd.2009.08.0137 20803388

[31] Comparison of a manual wheelchair designed and produced in Mexico to a wheelchair produced in China based on ISO testing and clinician and user feedback [Internet]. [cited 2015 Mar 23]. Available from: http://www.resna.org/sites/default/files/legacy/conference/proceedings/2013/WheeledMobility/Student Scientific/Toro.html

[32] Wang H, Liu H-Y, Pearlman J, Cooper RRA, Jefferds A, Connor S, et al Relationship between wheelchair durability and wheelchair type and years of test. Disabil Rehabil Assist Technol [Internet]. Informa UK Ltd London, UK; 2010 1;5(5):318–22. Available from: http://www.ncbi.nlm.nih.gov/pubmed/20131972 10.3109/17483100903391137 20131972

[33] Zipfel E, Cooper RA, Pearlman J, Cooper R, McCartney M. New design and development of a manual wheelchair for India. Disabil Rehabil. 2007 1;29(11–12):949–62. 10.1080/09638280701240672 17577729

[34] Rentschler AJ, Cooper RA, Boninger ML, Fitzgerald SG. Using Stability and Fatigue Strength Testing When Choosing a Manual Wheelchair. In: Proceedings of the RESNA 2001 Annual Conference. Reno, NV; 2001. p. 355–7.

[35] Kwarciak AM, Cooper RA, Ammer WA, Fitzgerald SG, Boninger ML, Cooper R. Fatigue testing of selected suspension manual wheelchairs using ANSI/RESNA standards. Arch Phys Med Rehabil [Internet]. 2005 1 [cited 2015 Feb 18];86(1):123–9. Available from: http://www.ncbi.nlm.nih.gov/pubmed/15641002 10.1016/j.apmr.2003.11.038 15641002

[36] United States Agency for International Development. United States Agency for International Development. 2016 10.1242/dev.135285

[37] World Health Organization. Global Cooperation on Assistive Technology (GATE). 2014.

[38] Pearlman J, Cooper R. Editorial. African J Disabil. 2017 Jan;6(0):3. 10.4102/ajod.v6i0.423 PMC5675921 29134180

[39] United Nations. Convention on the Rights of Persons with Disabilities. 2006. 10.1515/9783110208856.203 18348362

[40] Borg J, Khasnabis C. WHO Guidelines on the provision of manual wheelchairs in less-resourced settings Disabil Rehabil [Internet]. World Health Organization; 2008 [cited 2015 Jul 21]; Available from: http://www.who.int/disabilities/publications/technology/wheelchairguidelines/en/#.Va2cj8BW0ok.mendeley 23785745

[41] Mhatre A, Martin D, McCambridge M, Reese N, Sullivan M, Schoendorfer D, et al Developing product quality standards for wheelchairs used in less-resourced environments. African J Disabil. 2017 1;6(0):15 pages. 10.4102/ajod.v6i0.288 PMC5594264 28936410

[42] International Organization for Standardization. ISO—ISO Standards—ISO/TC 173/SC 1—Wheelchairs [Internet]. 2014 [cited 2015 Mar 9]. Available from: http://www.iso.org/iso/home/standards_development/list_of_iso_technical_committees/iso_technical_committee.htm?commid=53792

[43] ISO Technical Committee 173 SubCommittee 1 Working Group 1. ISO/AWI 7176–32—Weelchair—Part 32: Standard Practice for Wheelchair Castor Durability Testing [Internet]. 2019 [cited 2019 Apr 23]. Available from: https://www.iso.org/standard/77566.html

[44] Cooper R, Star J, Heil T. Development of a new ISO wheelchair two-drum tester. In: Proceedings of the Annual International Conference of the IEEE Engineering in Medicine and Biology Society Volume 13: 1991. IEEE; 1991. p. 1867–8.

[45] International Organization for Standardization. ISO—ISO Standards—ISO/TC 173/SC 1—Wheelchairs. 2014.

[46] Partners for Care. Partners for Care | Delivering health and hope in East Africa [Internet]. 2019 [cited 2019 Apr 23]. Available from: https://www.partnersforcare.org/

[47] Gulf Coast Data Concepts L. GCDC X16-1D Usb-Accelerometer 3-axis Data Recorder. 2016.

[48] ASTM International. ASTM B117 [Internet]. 2016. Available from: https://www.astm.org/Standards/B117.htm

[49] ASTM International. ASTM G1–03(2017)e1 Standard Practice for Preparing, Cleaning, and Evaluating Corrosion Test Specimens [Internet]. 2017 [cited 2019 Mar 31]. Available from: https://www.astm.org/Standards/G1

[50] Auto Technology Company. Auto Technology Salt Fog Cabinet.

[51] Mhatre A, Pearlman JL, Lachell S. Development, reliability, and piloting of a wheelchair caster failure inspection tool (C-FIT). Disabil Rehabil Assist Technol [Internet]. 2018; Available from: 10.1080/17483107.2018.1554714 30729825

[52] Pearlman J, Brienza D, Mhatre A, Ott J. Use of Performance Standards in Wheelchair Selection. In: International Seating Symposium 2019 [Internet]. 2019. Available from: https://www.seatingsymposium.us/event-schedule/event/3

[53] Tolerico ML, Ding D, Cooper RA, Spaeth DM, Fitzgerald SG, Cooper R, et al Assessing mobility characteristics and activity levels of manual wheelchair users. J Rehabil Res Dev [Internet]. 2007 1 [cited 2015 Feb 18];44(4):561–71. Available from: http://www.ncbi.nlm.nih.gov/pubmed/18247253 10.1682/jrrd.2006.02.0017 18247253

[54] Ma Y, Li Y, Wang F. The atmospheric corrosion kinetics of low carbon steel in a tropical marine environment. Corros Sci. Elsevier; 2010;52(5):1796–800.

[55] Natesan M, Venkatachari G, Palaniswamy N. Corrosivity and durability maps of India. Corros Prev Control. Beaconsfield [etc] Scientific Surveys Ltd.; 2005;52(2):43–55.

[56] Mohan PS, Natesan M, Sundaram M, Balakrishnan K. Atmospheric corrosion at different locations in South India. Bull Electrochem. Central Electrochemical Research Institute; 1996;12(1):91–2.

[57] Syed S. Atmospheric corrosion of hot and cold rolled carbon steel under field exposure in Saudi Arabia. Corros Sci. Elsevier; 2008;50(6):1779–84.

[58] Dean SW, Delgadillo GH-D, Bushman JB. Marine corrosion in tropical environments. In ASTM; 2000.

[59] Castaño JG, Botero CA, Restrepo AH, Agudelo EA, Correa E, Echeverría F. Atmospheric corrosion of carbon steel in Colombia. Corros Sci. Elsevier; 2010;52(1):216–23.

[60] Morales J, Martin-Krijer S, Díaz F, Hernández-Borges J, González S. Atmospheric corrosion in subtropical areas: influences of time of wetness and deficiency of the ISO 9223 norm. Corros Sci. Elsevier; 2005;47(8).

[61] Houska C. Metals for Corrosion Resistance. The Construction Specifier. Alexandria, VA; 2000.

[62] Karuu S. The study of the effects of some Kenyan soils on the corrosion of the underground pipes [Internet]. 1989. Available from: http://erepository.uonbi.ac.ke:8080/xmlui/handle/123456789/21932

[63] Mukherjee G, Samanta A. Wheelchair charity: a useless benevolence in community-based rehabilitation. Disabil Rehabil. Informa UK Ltd UK; 2005 5;27(10):591–6. 10.1080/09638280400018387 16019868

[64] Liu H, Pearlman J, Cooper R, Hong E, Wang H, Salatin B, et al Evaluation of aluminum ultralight rigid wheelchairs versus other ultralight wheelchairs using ANSI/RESNA standards. J Rehabil Res Dev. 2010 1;47(5):441–55. 10.1682/jrrd.2009.08.0137 20803388

[65] Cooper RA, Boninger ML, Rentschler A. Evaluation of selected ultralight manual wheelchairs using ANSI/RESNA standards. Arch Phys Med Rehabil. 1999 4;80(4):462–7. 10.1016/s0003-9993(99)90287-3 10206612

[66] Gebrosky B, Pearlman J, Cooper RA, Cooper R, Kelleher A. Evaluation of lightweight wheelchairs using ANSI/RESNA testing standards. J Rehabil Res Dev. Rehabilitation Research and Development Service; 2013 1;50(10):1373–89. 10.1682/JRRD.2012.08.0155 24699973

[67] Kwarciak AM, Cooper RA, Ammer WA, Fitzgerald SG, Boninger ML, Cooper R. Fatigue testing of selected suspension manual wheelchairs using ANSI/RESNA standards. Arch Phys Med Rehabil. 2005 1;86(1):123–9. 10.1016/j.apmr.2003.11.038 15641002

[68] Mhatre A. Development and validation of a wheelchair caster testing protocol [Internet]. 2018 [cited 2018 Sep 10]. Available from: http://d-scholarship.pitt.edu/33876/

[69] Sullivan M, Pearlman J, Mhatre A, Martin D, McCambridge M. Design Considerations for Wheelchairs used in Adverse Conditions. 2016.

