# Supplementary material for: Design and evaluation of a laboratory-based wheelchair castor testing protocol using community data
Source: PLoS One. 2020 Jan 10;15(1):e0226621. doi: 10.1371/journal.pone.0226621 (PMC6953824; doi:10.1371/journal.pone.0226621)
Supplement: S1 File — (DOCX) [file pone.0226621.s001.docx]

# Supporting Information

Stem bolt (vertical spindle or pintle) fracture failures in the community as shown in Fig S1 can be hazardous as the user may fall out of the chair during propulsion and other parts of the wheelchair may break. The bending stress analysis was conducted for this failure. The minimal stress that contributes to fatigue for medium strength steels was found using their S-N curves [1]. Most manufacturers use the 8.8 grade bolt (medium carbon steel, quenched and tempered) whose fracture strength or the ultimate tensile strength is 830 MPa [2]. Based on the S-N curves, stresses above 50% of fracture strength cause fatigue and hence, stresses above 415 MPa were considered for shock validation.

Accelerations (Fig 9) recorded in this study were converted into stress using bending moment and stress equations listed below and accelerations responsible for fatigue (bending stress > 415 MPa) were filtered for shock correlation. Since vertical reaction force due to user weight is responsible for 80-95% of bending stress in the stem bolt, only vertical accelerations were included for the static analysis. Dynamic analysis of the castor rolling over an obstacle was not pursued in this study.

$Bending Moment={(Mass}_{User}+{Mass}_{Wheel}+{Mass}_{Fork}) x Vertical Acceleration x Caster Trail$

$$Bending Stress=\frac{Bending Moment x Distance of farthest point from neutral axis}{Moment of Interia of the Stem Bolt}$$

***INSERT S1 Fig HERE***

**S1 Fig. Stem bolt fracture failure**

# References

1. Boyer HE. Atlas of fatigue curves. Asm International; 1985.

2. ASTM International. ASTM F568M. 2007.
