# Supplementary figures and images for: Design and evaluation of a laboratory-based wheelchair castor testing protocol using community data
Source: PLoS One. 2020 Jan 10;15(1):e0226621. doi: 10.1371/journal.pone.0226621 (PMC6953824; doi:10.1371/journal.pone.0226621)

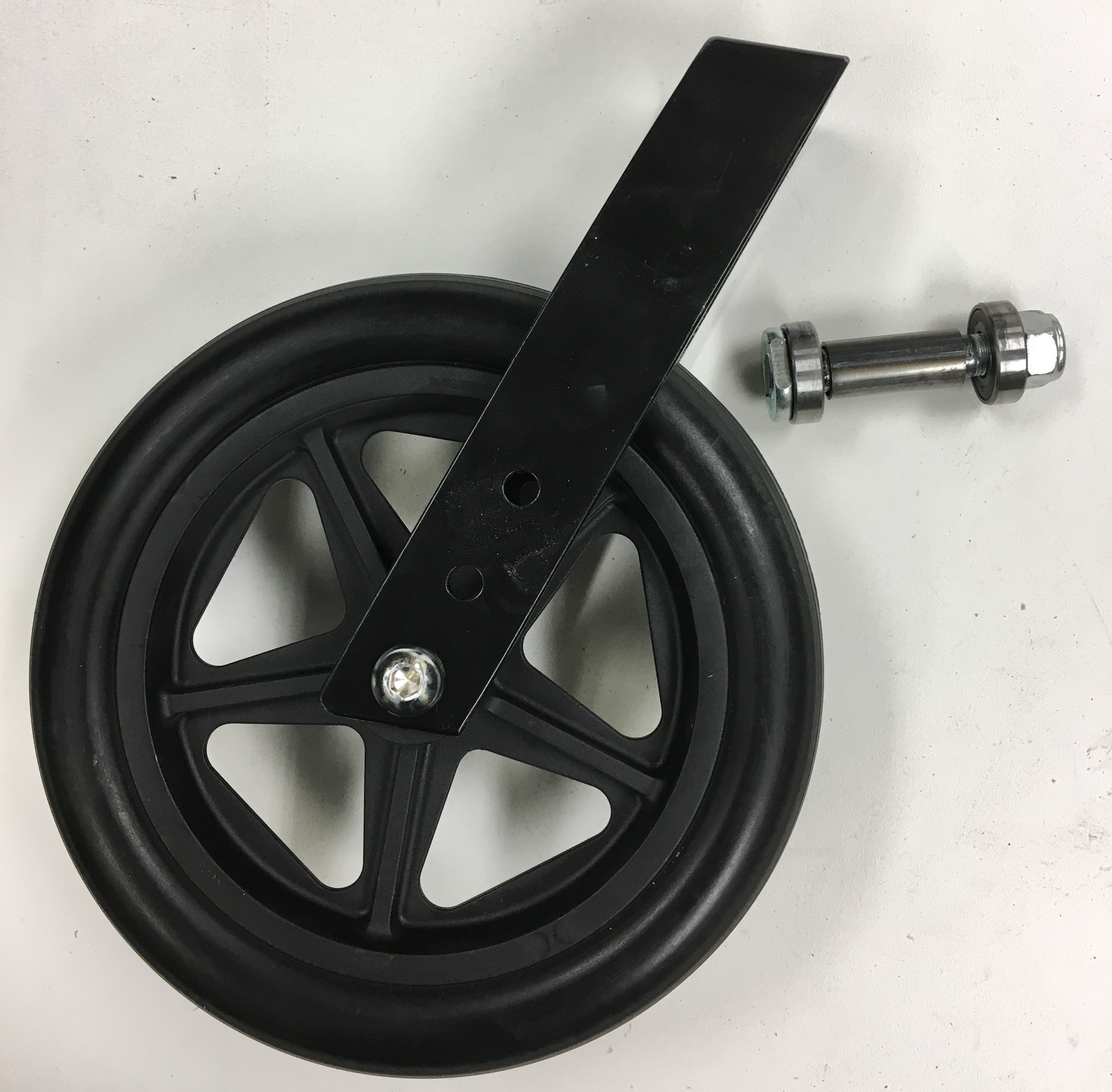

Supplement: S1 Fig — (TIFF) [file pone.0226621.s002.tiff]
